# Supplementary material for: From sequence to enzyme mechanism using multi-label machine learning
Source: BMC Bioinformatics. 2014 May 19;15:150. doi: 10.1186/1471-2105-15-150 (PMC4229970; doi:10.1186/1471-2105-15-150)
Supplement: Additional file 2 — Java code of ml2db. Additional file ml2db_code.tar.gz contains the Java source code to run the multi-label machine learning experiments and save the results to database. The code’s Javadoc is included. [file 1471-2105-15-150-S2.zip › additional file 2/ml2db/ml2db/doc/main/ml2db/weka/AttributeFactory.html]

AttributeFactory


JavaScript is disabled on your browser.


- Overview
- Package
- Class
- Use
- Tree
- Deprecated
- Index
- Help

- Prev Class
- Next Class

- Frames
- No Frames

- All Classes

- Summary:
- Nested |
- Field |
- Constr |
- Method

- Detail:
- Field |
- Constr |
- Method


main.ml2db.weka

## Class AttributeFactory

- java.lang.Object
- - main.ml2db.weka.AttributeFactory

- Direct Known Subclasses:
  :   MulanAttributeFactory

  ---

    

  ```
  public class AttributeFactory
  extends java.lang.Object
  ```

  The attribute factory produces binary and numeric Weka attributes.
  A nominal attribute is an attribute with a set of two or more possible string
  values. A binary attribute is a special nominal attribute with only two
  values: yes/no (or present/absent or zero/one). The attribute name often
  represents the characteristic that can be present or absent. Example: if the
  attribute name is a protein domain, the value yes/no represents whether the
  domain is present or absent in that protein sequence (instance). A numeric
  attribute contains numerical values (either integers or reals).
  Class attributes are generated as nominal.
  2013-03-01 "If it quacks, it's a duck" approach to attribute type. If the
  values are all "1", create NOMINAL {0,1} attribute with a default "0" value.
  If not, create a NUMERIC attribute with default "?" (unknown).

  Version:
  :   20 Feb 2008

  Author:
  :   Luna De Ferrari ldf(at)st-andrews.ac.uk

- - ### Field Summary

    Fields

    | Modifier and Type | Field and Description |
    | --- | --- |
    | `static java.lang.String` | `BINARY_ATTRIBUTE_ABSENT_VALUE` |
    | `static java.lang.String` | `BINARY_ATTRIBUTE_PRESENT_VALUE` |
    | `static java.lang.String` | `DUMMY_VALUE` |
    | `static java.lang.String` | `NOMINAL` |
    | `static java.lang.String` | `NUMERIC` |
    | `static java.lang.String` | `UNKNOWN` |
  - ### Constructor Summary

    Constructors

    Constructor and Description

    `AttributeFactory(DataSetGenerator dataSetGenerator)`

    Takes the attribute names from the dataset generator object.
  - ### Method Summary

    Methods

    | Modifier and Type | Method and Description |
    | --- | --- |
    | `static boolean` | `attributeIsValid(weka.core.Attribute attribute)` Check if an attribute is valid (not null, non-empty name) |
    | `void` | `createAttributes()` Generate the set of attributes |
    | `weka.core.Attribute` | `generateBinaryAttribute(java.lang.String attributeName)` Generates a binary attribute |
    | `weka.core.Attribute` | `generateNominalAttribute(java.lang.String attributeName, java.util.Vector<java.lang.String> values)` Generate a nominal attribute (with a list of possible values). |
    | `weka.core.Attribute` | `generateNominalAttributeWithDummy(java.lang.String attributeName, java.util.Set<java.lang.String> classValues)` Generate a nominal attribute (with a list of possible values). |
    | `weka.core.Attribute` | `generateNumericAttribute(java.lang.String attributeName)` Generates a numeric attribute |
    | `java.lang.String` | `getAttributeAbsentOption()` private void generateZeros() { double[] zeros = new double[this.getAttributes().size()]; Arrays.fill(zeros, 0.0); m\_zeros = zeros; } |
    | `weka.core.Attribute` | `getAttributeByName(java.lang.String name)` |
    | `java.lang.String` | `getAttributePresentOption()` |
    | `java.util.Vector<weka.core.Attribute>` | `getAttributes()` |
    | `java.util.Set<java.lang.String>` | `getAttributesNamesList()` |
    | `java.util.TreeMap<java.lang.String,java.lang.String>` | `getAttributesNameTypeMap()` |
    | `java.util.Vector<java.lang.String>` | `getBinaryAttributeValuesSet()` Get the possible attribute values for a yes/no attribute. |
    | `weka.core.Attribute` | `getClassAttribute()` Get last attribute as class attribute |
    | `DataSetGenerator` | `getDataSetGenerator()` |
    | `java.util.TreeMap<java.lang.String,weka.core.Attribute>` | `getNameAttributeMap()` |
    | `double[]` | `getZeros()` |
    | `void` | `intialise()` |
    | `void` | `setAttributesNameTypeMap(java.util.TreeMap<java.lang.String,java.lang.String> m_attributesNameTypeMap)` |

    - ### Methods inherited from class java.lang.Object

      `equals, getClass, hashCode, notify, notifyAll, toString, wait, wait, wait`

- - ### Field Detail


    - #### BINARY\_ATTRIBUTE\_ABSENT\_VALUE

      ```
      public static final java.lang.String BINARY_ATTRIBUTE_ABSENT_VALUE
      ```

      See Also:
      :   Constant Field Values


    - #### BINARY\_ATTRIBUTE\_PRESENT\_VALUE

      ```
      public static final java.lang.String BINARY_ATTRIBUTE_PRESENT_VALUE
      ```

      See Also:
      :   Constant Field Values


    - #### UNKNOWN

      ```
      public static final java.lang.String UNKNOWN
      ```

      See Also:
      :   Constant Field Values


    - #### NOMINAL

      ```
      public static final java.lang.String NOMINAL
      ```

      See Also:
      :   Constant Field Values


    - #### NUMERIC

      ```
      public static final java.lang.String NUMERIC
      ```

      See Also:
      :   Constant Field Values


    - #### DUMMY\_VALUE

      ```
      public static final java.lang.String DUMMY_VALUE
      ```

      See Also:
      :   Constant Field Values
  - ### Constructor Detail


    - #### AttributeFactory

      ```
      public AttributeFactory(DataSetGenerator dataSetGenerator)
      ```

      Takes the attribute names from the dataset generator object.

      Parameters:
      :   `dataSetGenerator` -
  - ### Method Detail


    - #### createAttributes

      ```
      public void createAttributes()
      ```

      Generate the set of attributes


    - #### generateBinaryAttribute

      ```
      public weka.core.Attribute generateBinaryAttribute(java.lang.String attributeName)
      ```

      Generates a binary attribute

      Parameters:
      :   `attributeName` - the attribute name

      Returns:
      :   the weka nominal attribute + a binary values set such as {0,1}


    - #### generateNominalAttribute

      ```
      public weka.core.Attribute generateNominalAttribute(java.lang.String attributeName,
                                                 java.util.Vector<java.lang.String> values)
      ```

      Generate a nominal attribute (with a list of possible values).

      Parameters:
      :   `attributeName` - the attribute name

      Returns:
      :   the weka attribute


    - #### generateNominalAttributeWithDummy

      ```
      public weka.core.Attribute generateNominalAttributeWithDummy(java.lang.String attributeName,
                                                          java.util.Set<java.lang.String> classValues)
      ```

      Generate a nominal attribute (with a list of possible values). Inserts a
      dummy value as first element to solve the problem at
      http://weka.wikispaces.com/ARFF+%28stable+version%29

      Parameters:
      :   `attributeName` - the attribute name

      Returns:
      :   the weka attribute


    - #### generateNumericAttribute

      ```
      public weka.core.Attribute generateNumericAttribute(java.lang.String attributeName)
      ```

      Generates a numeric attribute

      Parameters:
      :   `attributeName` - the attribute name

      Returns:
      :   the weka numeric attribute


    - #### getAttributeAbsentOption

      ```
      public java.lang.String getAttributeAbsentOption()
      ```

      private void generateZeros() { double[] zeros = new
      double[this.getAttributes().size()]; Arrays.fill(zeros, 0.0); m\_zeros =
      zeros;
      }


    - #### getAttributeByName

      ```
      public weka.core.Attribute getAttributeByName(java.lang.String name)
      ```


    - #### getAttributePresentOption

      ```
      public java.lang.String getAttributePresentOption()
      ```


    - #### getAttributes

      ```
      public java.util.Vector<weka.core.Attribute> getAttributes()
      ```


    - #### getAttributesNamesList

      ```
      public java.util.Set<java.lang.String> getAttributesNamesList()
      ```


    - #### getAttributesNameTypeMap

      ```
      public java.util.TreeMap<java.lang.String,java.lang.String> getAttributesNameTypeMap()
      ```


    - #### getBinaryAttributeValuesSet

      ```
      public java.util.Vector<java.lang.String> getBinaryAttributeValuesSet()
      ```

      Get the possible attribute values for a yes/no attribute. Beware!: the
      option "no" has to be listed as first option for the sparse ARFF format
      to work! http://weka.wikispaces.com/ARFF+%28stable+version%29

      Returns:
      :   The vector with the two values, for example: ["no", "yes"] .


    - #### getClassAttribute

      ```
      public weka.core.Attribute getClassAttribute()
      ```

      Get last attribute as class attribute

      Returns:
      :   a Weka `Attribute`


    - #### getDataSetGenerator

      ```
      public DataSetGenerator getDataSetGenerator()
      ```


    - #### getNameAttributeMap

      ```
      public java.util.TreeMap<java.lang.String,weka.core.Attribute> getNameAttributeMap()
      ```


    - #### getZeros

      ```
      public double[] getZeros()
      ```

      Returns:


    - #### intialise

      ```
      public void intialise()
      ```


    - #### setAttributesNameTypeMap

      ```
      public void setAttributesNameTypeMap(java.util.TreeMap<java.lang.String,java.lang.String> m_attributesNameTypeMap)
      ```


    - #### attributeIsValid

      ```
      public static boolean attributeIsValid(weka.core.Attribute attribute)
      ```

      Check if an attribute is valid (not null, non-empty name)

      Parameters:
      :   `attribute` -

      Returns:
      :   true if the attribute is valid (not null, non-empty name), false
          otherwise


- Overview
- Package
- Class
- Use
- Tree
- Deprecated
- Index
- Help

- Prev Class
- Next Class

- Frames
- No Frames

- All Classes

- Summary:
- Nested |
- Field |
- Constr |
- Method

- Detail:
- Field |
- Constr |
- Method
